# Challenges and changing roles for medical journals in the cyberspace age: Electronic pre-prints and e-papers

**DOI:** 10.2196/jmir.1.2.e9

**Published:** 1999-12-31

**Authors:** Gunther Eysenbach

*"We are in the business of revealing, not suppressing information."*

J. P. Kassirer, N. Eng. J. Med. 327, 1238 (1992).

In May 1999, the Director of the US National Institutes of Health (NIH), Harold Vermus, proposed a project, then dubbed "E-biomed" [1] (now called "PubMed Central" [2]). In this proposal, the National Institutes of Health - through the National Center for Biotechnology Information, a component of the National Library of Medicine - proposed to establish an Internet-accessible database of full electronic papers, which would be submitted directly by their authors. The original E-biomed plan [1] envisaged two sections in this system: one that allowed authors to submit papers which would only be electronically released after going through peer-review by editorial boards; and another one that allowed authors to publish electronic papers directly, without any peer-review, but with some minimal filtering in the form of requiring "approval by two individuals with appropriate credentials (...) to provide protection of the database from extraneous or outrageous material."

The proposal was embraced in numerous articles and editorials [3,4]; but several proponents of the traditional scientific publishing industry, especially the more conservative journals such as the New England Journal of Medicine (NEJM) uttered concerns, especially concerning the latter, non-peer-reviewed part of the system. Arnold S. Relman argued in a NEJM editorial that any "system that allowed immediate electronic publication of new clinical studies without the usual careful process of peer review and revision would be risky," [5] and argued that a "virtual community of experts and users could not provide practitioners with the kind of assistance they receive from the reports, reviews, and commentary found in high-quality peer-reviewed journals" [6].

Does this sound familiar? Indeed, the argument that peer-review is the only means of protecting the public from erroneous and thus potentially harmful research had been brought forward 30 years previous by former NEJM editor Franz Ingelfinger, who at that time announced the policy that a manuscript could not be accepted if it had previously been published elsewhere. Many journals have adopted this so-called Ingelfinger rule [1]; some of them, for example Science and the NEJM, have extended this rule into cyberspace, in that they will promptly reject manuscripts if they have been "published" on the World Wide Web: "posting a manuscript, including its figures and tables, on a host computer to which anyone on the Internet can gain access will constitute prior publication" [2]. This leads to the apparent paradox that articles which were published on the Web for the very purpose of open peer-review and in an effort to improve the manuscript are routinely rejected by these journals, with the argument that the public shall in this manner be protected from non-peer-reviewed, low-quality information. It shows the paternalistic concern of these journals, their belief that the public cannot discriminate between the different levels of credibility of a manuscript. Another explanation for this unwillingness to embrace electronic advance publication could be that traditional paper journals attempt to preserve their priority, newsworthiness, and exclusive access to research papers (which has long been a guarantee for circulation, attention, and paid advertisements for their journals). However, this race of traditional journals against the Internet for priority and exclusivity of research reports cannot be won by the journals.

Scholarly journals were originally established to serve scientists as a tool for communication. The Internet has the potential to further improve the communication process and perhaps - by fostering a broad and immediate debate - the quality of manuscripts. Systems such as the British Medical Journal (BMJ) e-print server and the original E-biomed proposal have the potential to make an article visible to experts before it is published in traditional print journals. It seems that any non-peer-reviewed paper published on the Web should be seen as a "virtual conference", thus papers presented in such a context on the Internet should be treated as papers presented at scientific meetings, for which the Ingelfinger rule is waived as well.

The NEJM editorial predicted that there would be "probably disastrous effects of E-biomed on journals" and frankly admitted the commercial concern that "a flourishing E-biomed system would very likely reduce the submissions, paid circulation, and income of most clinical journals enough to threaten their survival" [5]. However, I would argue that *every medical journal which can be replaced by an e-print server deserves to be* (in analogy to Warner Slacks saying that "any physician who can be replaced by a computer deserves to be" [7]).

Communication technology has changed profoundly since the times of Ingelfinger. These days, information is often first published on the Web and sometimes read by millions of users before printed journals can cover the story, which has perhaps most impressively been demonstrated by events such as the publication of the Starr Report on the Web [8]. In these cases, readers don't buy newspapers and journals because of their newsworthiness, but because of their in-depth analyses and comments.

A similar development in science seems to be inevitable and desirable. Medical journals - at least general medical journals - should give up their aim of being the primary and sole source of scientific information, but shift their aim toward acting as catalysts to get evidence-based medicine into practice. Their principal mission should not be newsworthiness, but putting "primary" information (which may have already been published on the Internet) into context and perspective, by evaluating, commenting, and weighting raw information. While the NEJM argues that the existence of non-peer-reviewed material on the Internet may confuse the public and pose a threat to traditional medical journals, we think that exactly the opposite will be true - that the more information is available, the more urgently traditional journals are needed to guide readers through this information jungle. The Internet will only make *bad* journals redundant - those journals which do little more than physically "publish" papers. The Internet will in the future become a huge library containing all the ingredients and information a researcher needs, but busy clinicians will still need journals such as the BMJ, Lancet, and even the NEJM to make the information digestible and to highlight the information which is relevant to clinical practice.

Perhaps even a new generation of journals will be established - journals which do not publish primary papers themselves, but which only evaluate, weight, comment, and put into context information that has been published elsewhere on the Web. Traditionally, the primary aim of journal publishers is to establish quality control mechanisms and to establish a reputation for the reliability of their information. In the near future, publishers - without putting any ink to paper or producing another medium - may get back to this fundamental truth of publishing - to be a credible source and to establish trust, to evaluate and describe information which perhaps is already published on the Internet. In fact, today we have two different meanings of "publishing" - one is the physical process of making a document public, the other is the process that implies establishing trust. While in traditional publishing, both of these processes were amalgamated into one single process, they are now separated; manuscripts may first be "published" on the Internet, but "establishing trust" may be a separate process and may have many different faces (e.g. favorable comments of colleagues, or a printed comment of a peer-reviewed journal pointing to a Web-published piece).

It is true, however, that there must be exceptions - certain manuscripts may for ethical reasons not be suitable for release on the Internet without previously having been peer-reviewed, if for example their results would raise false hopes among patients. However, these decisions should be made on an individual basis (perhaps made by the Institutional Review Board which approves the study) and should not be a basis for dismissing a whole new way of publishing.

Journals which do not change their editorial policies along these lines, allowing print-publication of material previously published on the Web for the sole purpose of review, and which continue to oppose and lobby against NIH plans to allow non-peer-reviewed material to be published in "PubMed Central", demonstrate that their primary concern is not to discourage public announcement of research findings which bypassed peer-review, but attempting to preserve their circulation by publishing exclusive reports before a broader audience has had the ability to access them. In doing so, they would cease to serve the research community but serve only the commercial interests of publishing companies. They would also demonstrate that they do not understand the Internet and that they do not understand that they are not longer in the business of revealing new information, but in making existing information understandable and useful for a broader audience.

Gunther Eysenbach, MD

Editor,

Journal of Medical Internet Research
